# Effect of premorbid beta-blockers on cardiac function and clinical outcomes in septic patients: a retrospective study

**DOI:** 10.62838/jccm-2026-0014

**Published:** 2026-04-30

**Authors:** Siyu Yang, Rongyao Feng, Xiangzhou Fang, Chaojie Wei

**Affiliations:** Zhongnan Hospital of Wuhan University, Wuhan Hubei, China

**Keywords:** sepsis, beta-blockers, septic cardiomyopathy, mortality, intensive care unit

## Abstract

**Background:**

Beta-blockers have been reported to exert potential beneficial effects in sepsis in recent years. However, their clinical application in sepsis remains limited due to concerns regarding hemodynamic impacts. This study aims to explore whether premorbid use of beta-blockers is associated with improvements in cardiac function and favorable clinical outcomes among patients with sepsis.

**Methods:**

This single-center, retrospective cohort study was conducted in the Intensive Care Unit (ICU) of a university-affiliated hospital. All patients diagnosed with sepsis admitted between August 2022 and March 2024 were enrolled. Exclusion criteria included age < 18 years, hospitalization duration < 48 hours, a history of severe underlying cardiac conditions, and incomplete clinical records. Primary outcomes included myocardial injury markers, echocardiographic parameters, and electrocardiographic indices to assess cardiac function. Secondary outcome was mortality.

**Results:**

Among 1005 septic patients, 228 had received premorbid beta-blockers. No significant difference in baseline disease severity was observed between the two groups. Patients with premorbid beta-blocker exposure had lower levels of cardiac troponin I (TnI, 87.9 [IQR, 23.4–306.0] vs 142.0 [IQR, 37.8–481.2]), lactic dehydrogenase (LDH, 274.0 [IQR, 175.0–496.0] vs 319.0 [IQR, 229.0–456.8]), and B-type natriuretic peptide (BNP, 267.9 [IQR, 118.1–1065.1] vs 509.3 [IQR, 184.8–1203.0]). Echocardiographic assessments revealed that premorbid beta-blockers were associated with a higher left ventricular ejection fraction (LVEF, 58% [IQR 52–60] vs 55% [IQR 50–60]). Additionally, premorbid beta-blockers were linked to lower 14-day (13.6% [IQR 9.1–18.1] vs 21.5% [IQR 18.6–24.4]), 28-day (17.5% [IQR 12.6–22.5] vs 27.4% [IQR 24.3–30.6]), and in-hospital (18.9% [IQR 13.7–24.0] vs 28.8% [IQR 25.6–32.0]) mortality rates.

**Conclusions:**

Among septic patients, premorbid beta-blockers are associated with preserved cardiac function and improved clinical outcomes. These findings highlight the need for prospective or randomized controlled trials to further explore the potential cardioprotective role of beta-blockers in sepsis.

## Background

Sepsis is widely defined as life-threatening organ dysfunction resulting from a dysregulated host response to infection [1]. Despite advances in clinical management, sepsis remains a leading cause of mortality in intensive care units (ICUs), with an estimated mortality rate of 26.7% [2]. Sepsis-induced cardiomyopathy (SIMD) is broadly characterized as an acute cardiac dysfunction syndrome unrelated to cardiac ischemia in septic patients [3], which can manifest in various forms, including primary myocardial cellular injury, systolic or diastolic impairment of the left and/or right ventricles, and inadequate cardiac output and oxygen delivery [3]. The reported prevalence of SIMD ranges from 10% to 70% [4], and its presence has been associated with a mortality risk of up to 70%, making it a key predictor of morbidity and mortality in this patient population [5]. Therefore, optimizing prevention and management strategies for SIMD is crucial for improving outcomes in sepsis.

The underlying mechanisms of sepsis-induced cardiac dysfunction have not been fully elucidated. Imbalanced inflammatory responses were initially proposed as a key contributor to SIMD [6,7], and oxidative stress has also been implicated as a critical factor [8]. Other potential mechanisms include pathogen-associated molecular patterns (PAMPs), mitochondrial dysfunction, altered calcium homeostasis, complement system activation, and myocardial edema [9,10,11,12]. Recently, excessive sympathetic system activation has gained increasing attention as a major factor associated with SIMD [13,14]. On one hand, sympathetic hyperactivation leads to excessive catecholamine release, which overstimulates cardiac beta-adrenergic receptors, potentially resulting in receptor desensitization and downregulation [15]. On the other hand, beta-adrenergic receptors are expressed on immune cells such as macrophages and lymphocytes, and continuous stimulation of these receptors may be associated with immune dysfunction [16]. Thus, beta-blockers, which inhibit excessive sympathetic activation, have been considered as a potential intervention in sepsis.

Beta-blockers have been reported to be associated with improved cardiac function in sepsis [17]. In animal models, beta-blocker administration has been linked to upregulation of cardiac beta-adrenergic receptors, as well as attenuation of cardiac inflammation and oxidative stress in sepsis [18,19]. Additionally, beta-blockers may be associated with reduced lymphocyte apoptosis and modulated lymphocyte differentiation in septic states [20]. In 2013, Morelli *et al*. conducted a randomized controlled trial (RCT) demonstrating that short-acting beta-blocker use during sepsis was associated with improved hemodynamic parameters and reduced mortality [21]. However, the clinical application of beta-blockers during sepsis is limited due to concerns about potential negative inotropic effects on myocardial contraction and hypotensive consequences. Few additional RCTs have been reported following the study by Morelli *et al*.

In response to these limitations, several observational studies have explored the association between premorbid beta-blockers and sepsis outcomes, yielding promising but inconsistent results [22]. Systematic reviews have concluded that the preliminary evidence regarding the role of premorbid beta-blockers in septic patients remains limited. Furthermore, the potential association between premorbid beta-blocker exposure and cardiac function in sepsis has not been fully characterized. Therefore, this retrospective cohort study was designed to explore the associations between premorbid beta-blockers and cardiac function, as well as clinical outcomes, in patients with sepsis.

## Methods

### Study design and data source

This single-center, retrospective study was conducted at Zhongnan Hospital of Wuhan University. The study population included patients with sepsis admitted to the ICU between August 2022 and March 2024. The study was approved by the Ethics Committee of Zhongnan Hospital of Wuhan University (Approval Number: 2024238K). Due to the retrospective nature of the study, informed consent was waived by the Ethics Committee. Clinical data of enrolled patients were retrospectively collected from the electronic medical record system. All procedures were performed in accordance with relevant guidelines and regulations.

### Population and variable

The study population consisted of patients admitted to the ICU for the first time. Eligibility criteria included a diagnosis of sepsis based on the Sepsis 3.0 criteria[1], and all patients received standard sepsis treatment. Patients were excluded if they met any of the following criteria: [1] age < 18 years; [2] hospitalization duration < 48 hours; [3] a history of severe underlying cardiac conditions, including myocardial infarction, congenital heart disease, severe valvular heart disease, and cardiomyopathy; [4] incomplete clinical records.

Included patients were divided into two groups based on the presence of premorbid beta-blockers. Previous studies exploring premorbid beta-blocker use in sepsis have varied in the definition of “premorbid” duration[23,24,25]; in this study, premorbid beta-blocker use was defined as initiation of therapy at least 30 days prior to ICU admission. Baseline information was collected to ensure comparability between the two cohorts. Disease severity was assessed using the maximum Sequential Organ Failure Assessment (SOFA) score and Acute Physiology and Chronic Health Evaluation II (APACHE II) score within the first 24 hours of admission.

### Study outcomes

The primary objective was to explore the association between premorbid beta-blockers and cardiac dysfunction in sepsis. Cardiac function was assessed using myocardial injury biomarkers, echocardiographic parameters, and electrocardiographic indices. The secondary objective was to compare mortality rates between patients with and without premorbid beta-blocker treatment.

### Statistical analysis

Data analysis was performed between July 10, 2024, and October 16, 2024. For missing baseline data, multiple imputation by predictive mean matching was used for continuous variables[26]. Continuous data were compared using the Mann-Whitney U test and presented as mean ± standard deviation (SD) or median (interquartile range [IQR]), as appropriate. Categorical variables were compared using the chi-square test. Differences in 28-day mortality were evaluated using the log-rank test. To account for potential confounding factors, the association between premorbid beta-blockers and mortality was assessed using logistic regression models adjusted for patient characteristics. All analyses were performed using SPSS version 27.0.1 (IBM, Chicago, IL, USA), and graphs were generated using GraphPad PRISM version 8.3.0 (San Diego, CA, USA). Statistical significance was set at p < 0.05. Additional and alternative statistical approaches are detailed in the Tables and Figures in the Supplementary materials.

## Results

### Baseline characteristics of subjects

Among 1640 patients meeting the sepsis diagnostic criteria, 57 were excluded due to short hospitalization duration, 530 had a pre-existing history of severe underlying cardiac conditions, and 48 had incomplete clinical data. A total of 1005 eligible patients were included in the retrospective cohort study, of whom 228 had received beta-blockers for at least 30 days prior to ICU admission, and 777 had no premorbid beta-blocker exposure. The majority (more than 90%) of patients in the beta-blocker group received selective β-receptor blockers, with metoprolol being the most commonly prescribed agent. Only one patient received propranolol, a non-selective β-receptor blocker (Table S1 in Supplementary materials). Due to incomplete documentation in the physician order entry system, specific dosages of beta-blockers could not be retrieved.

**Fig. 1.**
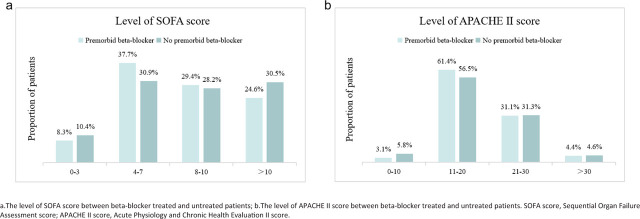
Levels of SOFA and APACHE II score among patients with or without premorbid beta-blocker.

Patient demographics and baseline clinical characteristics are summarized in Table 1, and disease severity (assessed by SOFA and APACHE II scores) is presented in Figure 1. The prevalence of hypertension (85.1% vs 65.4%, p < 0.001), coronary heart disease (18.4% vs 4.0%, p < 0.001), heart failure (8.8% vs 4.8%, p = 0.021), and arrhythmia (15.8% vs 5.7%, p < 0.001) was significantly higher in patients with premorbid beta-blockers. However, the two groups had similar sources of infection, pathogen distributions, vital signs, and hematological parameters at baseline.

**Table 1.** Patient Characteristics.

	**Overall population (N=1005, 100%)**	**Premorbid beta-blocker (N=228, 22.7%)**	**No premorbid beta-blocker (N=777, 77.3%)**	**P value**
Baseline characteristics				
Age, years, mean ± SD	70 ± 14	71 ± 11	69 ± 14	0.203
Gender (male), n, (%)	651 (64.8)	157 (68.9)	494 (63.6)	0.142
BMI (kg/m^2^), mean ± SD	22.8 ± 3.4	22.9 ± 3.6	22.9 ± 3.4	0.811

Coexisting conditions, n. (%)				
Hypertension	702 (69.9)	194 (85.1)	508 (65.4)	< 0.001
Coronary heart disease	73 (7.3)	42 (18.4)	31 (4.0)	< 0.001
Heart failure	57 (5.8)	20 (8.8)	37 (4.8)	0.021
Arrhythmia	80 (8.0)	36 (15.8)	44 (5.7)	< 0.001
Valvular heart disease	14 (1.3)	5 (2.2)	9 (1.2)	0.241
Diabetes	261 (26.0)	70 (30.7)	191 (24.6)	0.064
Chronic obstructive pulmonary disease	37 (3.7)	8 (3.5)	29 (3.7)	0.875
Chronic bronchitis	19 (1.9)	4 (1.8)	15 (1.9)	0.864
Bronchiectasis	10 (1.0)	4 (1.8)	6 (0.8)	0.190
Chronic kidney disease	89 (8.9)	24 (10.5)	65 (8.4)	0.313
Cerebrovascular disease	160 (15.9)	46 (20.2)	114 (14.7)	0.046
Malignancy	107 (10.6)	26 (11.4)	81 (10.4)	0.674
Autoimmune diseases	17 (1.7)	2 (0.9)	15 (1.9)	0.278

Source of sepsis, n. (%)				
Pulmonary	666 (66.3)	162 (71.1)	504 (64.9)	0.082
Intraabdominal	268 (26.7)	47 (20.6)	221 (28.4)	0.019
Urinary infections	137 (13.6)	33 (14.5)	104 (13.4)	0.674
Soft tissue infection	27 (2.7)	6 (2.6)	21 (2.7)	0.953
Bacteraemia	43 (4.3)	8 (3.5)	34 (4.4)	0.565
Other sources	21 (2.1)	4 (1.8)	17 (2.2)	0.678

Pathogen, n. (%)				
Gram-positive	142 (14.1)	34 (14.9)	108 (13.9)	0.700
Gram-negative	431 (42.9)	110 (48.2)	321 (41.3)	0.063
Anaerobic bacteria	7 (0.7)	1 (0.4)	6 (0.6)	0.074
Fungus	150 (14.9)	43 (18.9)	107 (13.8)	0.058
Virus	123 (12.2)	33 (14.5)	90 (11.6)	0.242
Unknown	290 (28.9)	63 (27.6)	227 (29.2)	0.643

Vital signs				
T (°C)	38.3 [37.7, 39]	38.2 [37.8, 38.9]	38.3 [37.7, 39]	0.556
HR (min^−1^)	104 [94, 117]	104 [95, 117]	103 [94, 117]	0.861
RR (min^−1^)	22 [20, 25]	22 [21, 26]	22 [20, 25]	0.251
MAP (mmHg)	71.3 [64.7, 77.0]	72.7 [66.7, 77.0]	71.0 [64.0, 77.0]	0.219

Vital lab data, median (IQR)				
WBC (10^9^/L)	11.0 [7.0, 16.7]	10.9 [7.3, 16.7]	11.1 [6.9, 16.9]	0.988
RBC (10^9^/L)	3.4 [2.8, 4.0]	3.4 [2.8, 4.1]	3.4 [2.8, 4.0]	0.497
PLT (10^9^/L)	141.0 [79.5, 217.5]	158.5 [93,229.8]	134.0 [74.5, 214.0]	0.038
HGB (g/L)	102.0 [83.2, 120.0]	102.0 [82.3, 121.8]	101.0 [83.7, 120.0]	0.959
CRP (mg/L)	99.0 [46.1, 160.0]	108.4 [52.2, 160.0]	97.9 [43.8, 160.0]	0.693
TBIL (umol/L)	16.2 [10.7, 30.1]	14.4 [10.3, 26.0]	16.6 [10.9, 31.9]	0.035
ALB (g/L)	29.7 [26.3, 33.6]	29.9 [26.4, 34.4]	29.7 [26.3, 33.3]	0.295
BNU (mmol/L)	11.9 [7.4, 20.0]	11.1 [7.1, 19.9]	11.9 [7.7, 20.0]	0.421
LAC (mmol/L)	3.3 [2.4, 4.5]	3.4 [2.4, 4.7]	3.3 [2.3, 4.7]	0.267
Severity, mean ± SD				
SOFA score	8.5 ± 3.8	8.3 ± 3.8	8.5 ± 3.8	0.519
APACHE II score	19.0 ± 6.2	18.8 ± 5.9	19.0 ± 6.2	0.677

T, temperature; HR, heart rate; RR, respiratory rate; MAP, mean arterial pressure; WBC, White Blood Cells; RBC, Red Blood Cells; PLT, Platelet; HGB, hemoglobin; CRP, hypersensitive C-reactive protein; TBIL, total bilirubin; ALB, albumin; BUN, blood urea nitrogen; LAC, lactic acid; SOFA score, Sequential Organ Failure Assessment score; APACHE II score, Acute Physiology and Chronic Health Evaluation II score; SD, standard deviation; IQR, interquartile range; Premorbid beta-blocker, patients with premorbid beta blocker therapy before septic episodes; No premorbid beta-blocker, patients without premorbid beta blocker therapy before septic episodes.

### Primary endpoint

Results of the primary outcome analysis are summarized in Table 2. Premorbid beta-blockers were associated with lower levels of myocardial injury markers, including troponin I (TnI, 87.9 [IQR, 23.4–306.0] vs 142.0 [IQR, 37.8–481.2], p = 0.004), B-type natriuretic peptide (BNP, 267.9 [IQR, 118.1–1065.1] vs 509.3 [IQR, 184.8–1203.0], p = 0.002), and lactic dehydrogenase (LDH, 274.0 [IQR, 175.0–496.0] vs 319.0 [IQR, 229.0–456.8], p = 0.022). Compared with patients without premorbid beta-blockers, those with premorbid beta-blocker therapy had a higher left ventricular ejection fraction (LVEF, 58.0 [IQR, 52.0–60.0] vs 55.0 [IQR, 50.0–60.0], p = 0.009).

**Table 2.** Comparison of myocardial injury markers, echocardiography and electrocardiogram abnormalities.

	**Premorbid beta-blocker (N=228, 22.7%)**	**No premorbid beta-blocker (N=777, 77.3%)**	**P value**
**Myocardial injury markers, median (IQR)**			
TnI (pg/ml)	87.9 (23.4–306.0)	142.0 (37.8–481.2)	0.004
BNP (pg/ml)	267.9 (118.1–1065.1)	509.3 (184.8–1203.0)	0.002
MYO (ng/ml)	209.8 (100.3–600.4)	283.6 (103.0–1046.0)	0.053
CK (U/L)	107.5 (43.3–253.0)	127.0 (48.0–307.0)	0.147
CKMB (U/L )	28.0 (13.0–97.0)	29.0 (16.0–63.0)	0.077
LDH (U/L)	274.0 (175.0–496.0)	319.0 (229.0–456.8)	0.022

**Echocardiographic parameters, median (IQR)**			
LVEF, (%)	58.0 (52.0–60.0)	55.0 (50.0–60.0)	0.009
LAD, (cm)	3.4 (3.0–3.9)	3.4 (3.1–3.9)	0.243
LVEDD, (cm)	4.3 (4.2–4.6)	4.5 (4.3–4.8)	< 0.001
RAD, (cm)	3.3 (3.2–3.6)	3.4 (3.3–3.7)	0.035
RVD, (cm)	3.4 (3.2–3.5)	3.4 (3.2–3.6)	0.007
PA, (cm)	2.3 (2.2–2.5)	2.3 (2.2–2.5)	0.092

**Electrocardiogram manifestations, no. (%)**			
Atrial fibrillation	41 (18.0)	113 (14.5)	0.205
Premature atrial contractions	27 (11.8)	127 (16.3)	0.097
Atrial flutter	7 (3.1)	26 (3.3)	0.837
Atrial tachycardia	3 (1.3)	16 (2.1)	0.469
Supraventricular tachycardia	3 (1.3)	7 (1.0)	0.579
Premature ventricular contractions	19 (8.3)	66 (8.5)	0.939
Ventricular tachycardia	2 (0.8)	10 (1.3)	0.616
Atrioventricular block	5 (2.2)	16 (2.1)	0.901
Bundle branch block	41 (18.0)	143 (18.4)	0.885

TnI, troponin I; BNP, B-type natriuretic peptide; MYO, myoglobin; CK, creatine kinase; CKMB,creatine kinase isoenzyme; LDH, lactic dehydrogenase; LVEF, left ventricular ejection fraction; LAD, left atrial diameter; LVEDD, left ventricular end diastolic diameter; RAD, right atrial diameter; RVD, right ventricular diameter; PA, pulmonary artery diameter; IQR, interquartile range. Premorbid beta-blocker, patients with premorbid beta blocker therapy before septic episodes; No premorbid beta-blocker, patients without premorbid beta blocker therapy before septic episodes.

Premorbid beta-blocker exposure was also associated with smaller left ventricular end-diastolic diameter (LVEDD, 4.3 [IQR, 4.2–4.6] vs 4.5 [IQR, 4.3–4.8], p < 0.001), right atrial diameter (RAD, 3.3 [IQR 3.2–3.6] vs 3.4 [IQR 3.3–3.7], p = 0.035), and right ventricular diameter (RVD, 3.4 [IQR 3.2–3.5] vs 3.4 [IQR 3.2–3.6], p = 0.007). The most common arrhythmias in septic patients included bundle branch block (18.0% vs 18.4%, p = 0.885), atrial fibrillation (18.0% vs 14.5%, p = 0.205), and premature atrial contractions (11.8% vs 16.3%, p = 0.097). No significant differences in electrocardiographic manifestations were observed between the two groups.

### Secondary endpoints

**Fig. 2.**
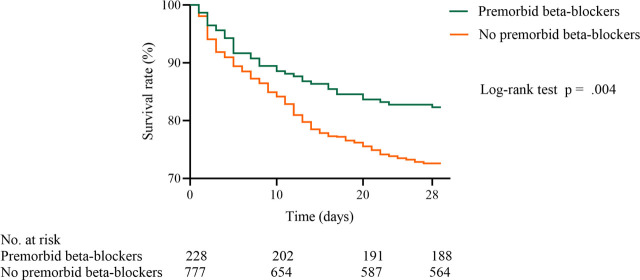
28-day survival rate for primary cohort.

Mortality outcomes were associated with premorbid beta-blockers in the unadjusted analysis (Table 3). After adjusting for potential confounders (Figure S1–S4 in the Supplementary materials), logistic regression models revealed that premorbid beta-blocker exposure was associated with lower 14-day (OR, 0.535; 95% CI, 0.345–0.830; p = 0.005), 28-day (OR, 0.516; 95% CI, 0.346–0.770; p = 0.001), and in-hospital (OR, 0.524; 95% CI, 0.535–0.776; p = 0.001) mortality rates. No significant difference in 7-day mortality was observed between the two groups (OR, 0.648; 95% CI, 0.385–1.090; p = 0.102). Additionally, the log-rank test showed that premorbid beta-blockers were associated with a significant reduction in 28-day mortality (hazard ratio, 0.61; 95% CI, 0.46–0.82; p = 0.004) (Figure 2).

**Table 3.** Analysis of mortality between premorbid beta-blocker treated and untreated patients.

	**Overall population (N=1005, 100%)**	**Premorbid beta-blocker (N=228, 22.7%)**	**No premorbid beta-blocker (N=777, 77.3%)**	**P value**
In-hospital mortality, (%), 95%CI	26.6 [23.8–29.3]	18.9 [13.7–24.0]	28.8 [25.6–32.0]	0.003
7-day mortality, (%), 95%CI	12.0 [10.0–14.1]	9.2 [5.4–13.0]	12.9 [10.5–15.2]	0.135
14-day mortality, (%), 95%CI	19.7 [17.2–22.2]	13.6 [9.1–18.1]	21.5 [18.6–24.4]	0.008
28-day mortality, (%), 95%CI	25.2 [22.5–27.9]	17.5 [12.6–22.5]	27.4 [24.3–30.6]	0.003

IQR, interquartile range; CI, confidence interval. Premorbid beta-blocker, patients with premorbid beta blocker therapy before septic episodes; No premorbid beta-blocker, patients without premorbid beta blocker therapy before septic episodes.

## Discussion

This retrospective cohort study explored the associations between premorbid beta-blockers and cardiac function, as well as clinical outcomes, in a large cohort of septic patients. Notably, despite a higher burden of cardiovascular comorbidities (including hypertension, coronary heart disease, heart failure, and arrhythmia) in patients with premorbid beta-blocker use, this group was associated with lower 14-day, 28-day, and in-hospital mortality rates compared with patients without premorbid beta-blocker exposure. Additionally, premorbid beta-blockers therapy was linked to favorable profiles of myocardial injury markers and echocardiographic parameters, suggesting potential cardioprotective associations in the context of sepsis. These findings add to the growing body of evidence regarding the role of beta-blockers in sepsis and highlight several important considerations for clinical practice and future research.

A critical observation derived from this study is the time-dependent pattern of the association between premorbid beta-blockers and mortality. No significant association with 7-day mortality was identified; however, favorable associations emerged at 14 days, persisted through 28 days, and remained evident during hospitalization. This time-dependent trend has not been fully characterized in prior literature and may be closely linked to the dynamic pathophysiological evolution of sepsis and SIMD. Sepsis is a highly dynamic syndrome characterized by an initial hyperinflammatory phase, which transitions to a prolonged phase of immune dysregulation and progressive organ dysfunction[27,28]. It is plausible that the potential benefits of premorbid beta-blockers become more pronounced in the later stages of sepsis, where sympathetic hyperactivation and persistent inflammation exert sustained adverse effects on cardiac function and overall prognosis. For instance, early sepsis is often dominated by acute hemodynamic instability and overwhelming inflammatory responses, which may mask any potential cardioprotective associations of beta-blockers. In contrast, during the subacute and chronic phases of sepsis, the cumulative effects of sympathetic overstimulation—such as beta-adrenergic receptor desensitization, myocardial remodeling, and ongoing oxidative stress—may be more effectively modulated by prior beta-blocker therapy. Consistent with this hypothesis, Fuchs *et al*. demonstrated that continuation of long-term beta-blocker therapy during the acute phase of severe sepsis is associated with improved survival up to 90 days (adjusted HR = 0.67; 95% CI 0.48–0.95; p = 0.003)[29]. Their findings align with our proposition that the beneficial effects of beta-blockers in sepsis may be delayed. Further investigations are therefore warranted to delineate temporal changes in the pathophysiology of sepsis and their implications for beta-blocker-associated outcomes.

The associations between premorbid beta-blockers and improved cardiac function markers in this study are consistent with several proposed mechanisms of SIMD pathophysiology. Myocardial injury markers such as TnI, LDH, and BNP are well-established indicators of cardiac dysfunction in sepsis [30,31,32,33,34,35]. TnI is a cardiac-specific marker, and its elevation in sepsis is often linked to myocardial cellular injury and SIMD [36], with higher levels associated with poorer outcomes [37]. The lower TnI levels observed in patients with premorbid beta-blocker exposure may be reflective of reduced myocardial injury in this group, potentially through modulation of pathways involved in sepsis-induced cardiac damage. Similarly, LDH, although non-specific, has been associated with in-hospital mortality in sepsis [31], and its lower levels in the beta-blocker group may indicate reduced overall organ injury, including cardiac injury. BNP is a marker of heart failure and ventricular stretch, and its elevation in sepsis is associated with SIMD severity and mortality [32,34,35]. The reduced BNP levels in patients with premorbid beta-blocker exposure may suggest attenuated ventricular dysfunction in this cohort.

Echocardiographic findings further support potential cardioprotective associations of premorbid beta-blockers. LVEF is a key measure of systolic function, and its preservation is associated with better outcomes in sepsis [38]. The higher LVEF observed in the beta-blocker group, even in the presence of more baseline cardiovascular comorbidities, is notable and may reflect a protective association against sepsis-induced systolic dysfunction. Additionally, the smaller LVEDD, RAD, and RVD in patients with premorbid beta-blocker exposure suggest potential associations with reduced ventricular and atrial remodeling in sepsis. Diastolic dysfunction and right ventricular dysfunction are common in sepsis [39,40], and while limited data on diastolic parameters were available in this study, the reduced chamber diameters may indirectly indicate preserved diastolic function or reduced ventricular overload. These echocardiographic findings are consistent with animal studies that have reported beta-blocker-associated reductions in myocardial inflammation, oxidative stress, and ventricular remodeling in sepsis [18,19]. It is possible that premorbid beta-blockers may induce adaptive changes in cardiac structure and function—such as upregulation of beta-adrenergic receptors or reduced myocardial fibrosis—that confer resilience against sepsis-induced injury [14].

The potential mechanisms underlying the observed associations between premorbid beta-blocker exposure and favorable outcomes in sepsis are likely multifactorial and may involve interactions between cardiac, immune, and inflammatory pathways. Beta-blockers are known to inhibit sympathetic hyperactivation, which is a key driver of SIMD [13,14]. By reducing catecholamine-mediated overstimulation of cardiac beta-adrenergic receptors, premorbid beta-blocker therapy may prevent or mitigate receptor desensitization and downregulation, thereby preserving cardiac contractile function during sepsis [15]. Additionally, beta-blockers have been shown to modulate inflammatory responses in sepsis [20,41], with potential reductions in pro-inflammatory cytokines and oxidative stress—both of which are critical contributors to myocardial injury [6,8]. Beta-blockers may also exert immunomodulatory effects by regulating lymphocyte function and apoptosis [20], which could influence the overall immune response to sepsis and reduce organ damage. These mechanisms are not mutually exclusive and may interact synergistically to contribute to the favorable associations observed in this study.

The findings of this study should be interpreted in the context of existing literature. Previous observational studies have reported inconsistent associations between premorbid beta-blocker use and sepsis outcomes[22,23,24,25,42]. For example, some studies have reported favorable mortality associations [43,44], while others have found no significant effects [45]. These discrepancies may be attributed to differences in study populations (e.g., varying baseline comorbidities, sepsis severity), definitions of premorbid beta-blocker use (e.g., duration of therapy, type of beta-blocker), and adjustment for confounding factors. The current study adds value by focusing on cardiac function outcomes, which have been less extensively explored in previous studies of premorbid beta-blockers in sepsis, and by including a large sample size with detailed assessment of myocardial markers and echocardiographic parameters. Additionally, the high proportion of selective β-receptor blockers in this study (predominantly metoprolol) is consistent with clinical practice, where selective agents are often preferred to minimize adverse effects [22,46]. The potential differences in associations between selective and non-selective beta-blockers in sepsis remain unclear, given the small number of non-selective beta-blocker users in this study.

Several clinical implications may be drawn from this study. First, the findings suggest that continuation of beta-blocker therapy in patients with pre-existing cardiovascular conditions who develop sepsis may be associated with favorable cardiac and mortality outcomes, despite concerns about potential hemodynamic risks. This is particularly relevant given that beta-blockers are commonly prescribed for chronic cardiovascular diseases, and their discontinuation during sepsis is a frequent clinical dilemma. Second, the time-dependent mortality association highlights the importance of long-term follow-up in sepsis studies, as early outcomes may not capture the full spectrum of treatment effects. Third, the potential cardioprotective associations of premorbid beta-blockers suggest that beta-blocker therapy could be a target for further research in sepsis prevention or management, particularly in patients at high risk of SIMD.

### Strengths and Limitations

It is important to acknowledge the limitations of this study when interpreting the findings. As a retrospective, single-center study, there is inherent potential for selection bias and unmeasured confounding. For example, patients with premorbid beta-blocker exposure had more cardiovascular comorbidities, which may have influenced treatment decisions and outcomes despite adjustment in regression models. Additionally, data on beta-blocker dosages, duration of therapy beyond 30 days, and adherence to therapy were not available, which may have impacted the observed associations. Furthermore, clinical data were limited to ICU admission, and information from the emergency department or general ward was not captured, which may have omitted important baseline or early sepsis data. Finally, the absence of a standardized dosage regimen for beta-blocker administration limits the clinical applicability and guiding value of the study's findings.

Despite these limitations, this study provides valuable insights into the potential associations between premorbid beta-blocker therapy and cardiac function, as well as mortality, in sepsis. Future research should focus on prospective, multicenter studies designed to confirm these findings and explore the underlying mechanisms. Randomized controlled trials comparing continuation versus discontinuation of beta-blocker therapy in septic patients with pre-existing beta-blocker use would be particularly valuable to establish causal relationships. Additionally, studies investigating the effects of different types and dosages of beta-blockers, as well as the optimal timing of therapy, could further refine clinical recommendations. Research into the time-dependent effects of beta-blockers in sepsis, including associations with different phases of sepsis pathophysiology, may also provide important mechanistic insights.

## Conclusion

In this retrospective cohort study of patients with sepsis, premorbid beta-blocker treatment was associated with an improved cardiac function and clinical outcomes. These results support the need for prospective or randomized clinical trials to confirm the cardioprotective effects of the administration of beta-blocker in sepsis.
